# The Neurocircuitry of Cannabis Cue Reactivity in Cannabis Use Disorder: A Functional Neuroimaging Study

**DOI:** 10.1016/j.bpsgos.2025.100638

**Published:** 2025-10-16

**Authors:** Valentina Lorenzetti, Hannah Sehl, Arush Honnedevasthana Arun, Eugene McTavish, Adam Clemente, Hannah Thomson, Marianna Quinones-Valera, Alexandra Gaillard, Emillie Beyer, Diny Thomson, Janna Cousijn, Izelle Labuschagne, Peter Rendell, Gill Terrett, Chao Suo, Lisa-Marie Greenwood, Victoria Manning, Govinda Poudel

**Affiliations:** a Neuroscience of Addiction and Mental Health Program, Healthy Brain and Mind Research Centre, School of Behavioural and Health Sciences, Faculty of Health Sciences, Australian Catholic University, Fitzroy, Victoria, Australia; b Addiction and Clinical Neurosciences Laboratory, Department of Health Sciences and Biostatistics, Swinburne University of Technology, Hawthorn, Victoria, Australia; c Turner Institute for Brain and Mental Health, School of Psychological Science, Monash University, Clayton, Victoria, Australia; d Neuroscience of Addiction Laboratory, Center for Substance Use and Addiction Research, Department of Psychology, Education and Child Studies, Erasmus University Rotterdam, Rotterdam, the Netherlands; e School of Behavioural & Health Sciences, Australian Catholic University, Fitzroy, Victoria, Australia; f School of Psychology, University of Queensland, Brisbane, Queensland, Australia; g School of Medicine and Psychology, Australian National University College of Health and Medicine, Australian National University, Canberra, Australia; h Monash Addiction Research Centre, Eastern Health Clinical School, Monash University, Box Hill, Victoria, Australia; i Turning Point, Eastern Health, Monash University, Melbourne, Victoria, Australia; j Mary MacKillop Institute for Health Research, Australian Catholic University, Melbourne, Victoria, Australia; k Braincast Neurotechnologies, Melbourne, Victoria, Australia

**Keywords:** Cannabis, Cannabis use disorder, Craving, Cue reactivity, fMRI, Neuroimaging

## Abstract

**Background:**

A common feature of cannabis use disorder (CUD) is an intense reactivity to cannabis cues, which are becoming increasingly visible due to the growth in its decriminalization, accessibility, and marketing of cannabis products. The brain’s automatic reactivity to cannabis cues can trigger craving and subsequent use. In this study, we aimed to test neural activity during cannabis cue reactivity in non–treatment-seeking individuals with moderate-to-severe CUD and past attempts to cut down/quit.

**Methods:**

The study examined 65 individuals with moderate-to-severe CUD and 43 control participants, with a functional magnetic resonance imaging cannabis cue-reactivity task and assessment of mental health and substance use as well as cognitive testing. Group differences in neural responses to cannabis cue reactivity were examined, adjusting for age and sex; correlations with cannabis use characteristics and mental health variables were assessed, accounting for recent substance use.

**Results:**

Compared with control participants, individuals with CUD showed greater brain activity during cannabis cue reactivity in the superior/middle occipital, medial/lateral orbitofrontal cortex, anterior/posterior cingulate, cerebellar, hippocampus, and middle temporal and lateral parietal cortices (*p* < .05; cluster *k* > 10, familywise error corrected). Greater occipital/cerebellar activity correlated with greater subjective arousal toward cannabis images and cannabis withdrawal scores, while anterior cingulate/inferior parietal activity negatively correlated with urinary level of 11-Nor-9-carboxy-Δ^9^-tetrahydrocannabinol:creatinine (*p*s < .05).

**Conclusions:**

Exposure to cannabis cues can elicit greater activity within salience evaluation/attention, motivation, and disinhibition pathways of addiction neurocircuitry in people with moderate-to-severe CUD, consistent with prominent neuroscientific theories of addiction and findings with other substances. Interventions that can suppress brain activity in salience and attention circuits during cannabis cue reactivity may help reduce craving and subsequent use.

## Plain Language Summary

People with cannabis use disorder (CUD) demonstrate brain reactivity to cannabis-related cues, which can increase cravings and lead to use. This study used real-time brain scans to examine how individuals with moderate-to-severe CUD respond to cannabis cues compared with people without CUD. People with a CUD showed greater activity in brain areas linked to attention, motivation, and disinhibition. This activity was also related to how strongly they craved cannabis. Future research is needed to tell us whether reducing brain reactivity to cannabis cues can help people with CUD manage cravings and avoid using cannabis.

Approximately 22 million people globally experience a cannabis use disorder (CUD) during their lifetime, representing around 1 in 4 people who use cannabis (1). Since 2019, there has been a 32% increase in the incidence of CUD and a 39% increase in CUD-related disability-adjusted life years, concomitant with accidental poisoning, schizophrenia, anxiety, depression, and road traffic injuries (2). CUD can be associated with significant adverse psychosocial outcomes, such as withdrawal, and a higher prevalence of anxiety, depression, and psychotic disorders (3). Interestingly, however, it has been reported that only a minority (∼13%) of individuals with CUD seek treatment (4). Most people who experience problems with cannabis use attempt to reduce or cease use without treatment. However, research suggests that unsuccessful quit attempts are common, with one study showing that 61% of people made a subsequent reattempt within 2 to 3 months of initiating daily monitoring of use (5). These statistics are concerning and highlight the need to uncover the mechanisms underlying CUD in individuals who continue consuming cannabis, despite attempts to reduce or cease use.

The experience of strong craving, i.e., an intense desire/preoccupation to use cannabis, which may be exacerbated after being exposed to cannabis-related cues (e.g., environmental cues such as images of cannabis, paraphernalia in a shop window, smelling the odor of cannabis at social gatherings, or internal sensations such as stress) (6), is a core feature contributing to continued cannabis use, relapse, or failed attempts to reduce cannabis use and predicting heavier use in CUD. As more jurisdictions decriminalize cannabis products globally, cannabis-related cues in the environment will become increasingly ubiquitous, triggering cravings and making it difficult for people who want to reduce or cease using cannabis. Cannabis cue reactivity has been ascribed to the alteration of brain function within selected brain reward pathways, including striatal and prefrontal (prefrontal cortex [PFC]) regions (e.g., anterior cingulate, middle frontal gyrus), parietal regions (posterior cingulate/precuneus), and additional brain areas (7).

Functional magnetic resonance imaging (fMRI) studies have examined brain activity in cannabis users during fMRI tasks using cannabis cue reactivity (8,9) [for a systematic review, see (10)]. These studies have shown increased brain activity in cannabis users during exposure to cannabis cues in regions involved in cognitive processes typically altered in CUD (8, 9, 10). Such cognitive domains and their underlying brain regions include reward processing (e.g., nucleus accumbens); habit formation/learning (e.g., striatum, hippocampus); motivation and disinhibition (e.g., PFC, orbitofrontal cortex [OFC], frontal medial cortex); and self-monitoring and awareness of environmental stimuli (e.g., precuneus) (8,9,11); as well as salience (e.g., occipital cortex and posterior cingulate cortex [PCC]) (8,9,12).

Overall, the fMRI evidence to date shows that cannabis use is associated with changes in partially overlapping brain pathways that are implicated in prominent neuroscientific theories of addiction (e.g., striatum, PFC, PCC) (7). Furthermore, this evidence shows that different functioning in cannabis users, in striatal, orbitofrontal, amygdala, occipital, and insular pathways implicated in neuroscientific theories of addiction (7), is associated with greater cannabis exposure and related problems, including craving, loss of control over use, grams used per month, and Δ^9^-tetrahydrocannabinol (THC) metabolites (10). Inconsistencies in altered brain activity in fMRI cannabis cue reactivity likely reflect methodological differences (e.g., heterogeneity across samples in levels of cannabis use) and limitations across studies. For example, only around one half of the studies included control groups (10), only a handful correlated brain function with metrics of cannabis exposure/problems (10), and few examined whether altered brain function in cannabis users is associated with confounds (e.g., nicotine and alcohol exposure, abstinence duration) (10). Furthermore, examination of fMRI cannabis cue reactivity in participants who meet criteria for moderate-to-severe CUD, are non–treatment seeking, and report having attempted to reduce or quit using cannabis (10) is relevant, because this represents a substantial proportion of people who use cannabis.

In this study, we aimed to overcome the limitations of the literature reported to date. The primary aim was to compare brain function during a cannabis cue-reactivity fMRI task, for the first time, in individuals meeting criteria for moderate-to-severe CUD and who self-report previous attempts to cut down or quit use, with that of nonusing control participants. Consistent with the emerging literature (13) and neurobiological theories of addiction (7), we hypothesized greater activity during cannabis versus neutral cues in the striatum, PFC (i.e., OFC, anterior cingulate cortex [ACC], medial frontal gyrus), and visual and parietal regions (i.e., precuneus, PCC) in participants in the moderate-to-severe CUD group compared with control participants. Our secondary aim was to explore associations between brain activity during cannabis cue reactivity and CUD symptom severity, subjective craving, and arousal ratings in response to cannabis cues, as well as additional metrics of cannabis exposure and mental health symptom scores, accounting for recent cannabis, alcohol, and cigarette use.

## Methods and Materials

The study is nested within a larger project where methods were preregistered (http://www.isrctn.com/ISRCTN76056942). The study was approved by the Human Research Ethics Committee at the Australian Catholic University (HREC: 2019-71H).

### Advertisement

Participants were recruited from the Melbourne metropolitan area, Australia, via public platforms (e.g., Facebook, flyers in the general community/university campuses). Study advertisements included a link that directed community members to a ∼25-minute online survey to screen participants using the study’s eligibility criteria.

### Participant Selection

Participants were neurologically healthy adults screened using key inclusion and exclusion criteria. Inclusion criteria in the cannabis use group included 1) meeting criteria for moderate-to-severe CUD based on the Structured Clinical Interview for DSM-5 Research Version, 2) daily/near-daily cannabis use over the past 12 months, and 3) attempted to quit or cut down at least once over the past 24 months. Additional inclusion and exclusion criteria and eligibility processes for all participants are detailed in the Supplement.

### Assessment Procedure

All assessments lasted approximately 4 to 6 hours and were conducted by trained researchers and students at Monash Biomedical Imaging in Clayton, Victoria, Australia. All participants gave written informed consent prior to participation. Assessment included questionnaires administered via Qualtrics Version XM (https://www.qualtrics.com), face-to-face semistructured interviews for substance use and mental health profiling, and cognitive testing (e.g., IQ) as detailed in the Supplement. The MRI scan included a structural T1-weighted image analyzed for registration purposes and an fMRI cannabis cue-reactivity task during which participants viewed cannabis images and visually matched control images. The assessment concluded with a debriefing and voucher reimbursement (i.e., Coles/Myers) of $100 for the control group and $150 for the CUD group. Additional reimbursement in the CUD group was to compensate participants for further research activities as part of the broader study. Participant data collection took place between October 2019 and July 2022.

### fMRI Cannabis Cue-Reactivity Task

**Figure 1:**
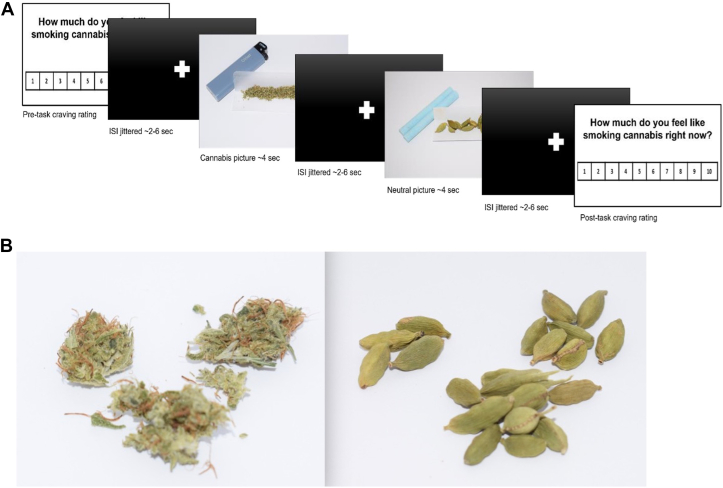
Example of functional magnetic resonance imaging cue-reactivity task parameters. **(A)***S*timulus presentation, whereby a craving rating was presented at the start and completion of the task asking participants to rate “How much do you feel like smoking cannabis right now?” on a scale of 1 to 10. A total of 60 images were presented with a varying duration in seconds of interstimulus intervals (ISIs) shown in the black screen with a centered white *“*+.*”***(B)***S*amples of a cannabis image (left) and of a neutral image.

An event-related cannabis cue-reactivity fMRI task (Figure 1) was designed to measure brain activity while participants viewed 30 cannabis-related images depicting paraphernalia and use behaviors and 30 neutral images of stationary items or cooking utensils. Each image was displayed for 4 seconds, with a fixation cross appearing between images for an average of 4 seconds (±2 seconds), and the task lasted for about 10 minutes. See the Supplement for additional details on the fMRI task and participant instruction.

### Assessment of Valence and Craving: Cannabis and Neutral Cues

Immediately before and after completion of the fMRI task, participants rated their arousal (1 = calm to 9 = excited) and affective valence (1 = unpleasant to 9 = pleasant) on a 9-point visual analog scale (VAS) and their cannabis cravings using the 10-point VAS question “how much do you feel like smoking cannabis right now?” (1 = not at all to 10 = extremely).

### fMRI Data Analysis

First-level analyses were run on MATLAB (version 2018a; The MathWorks, Inc.) with SPM12 (http://fil.ion.ucl.ac.uk/spm/) using a general linear model (GLM) to quantify the relationship between the observed event-related blood oxygen level–dependent (BOLD) signals and 2 regressors (i.e., cannabis and neutral images). The 6 motion estimates (i.e., translation and rotation on 3 axes: x, y, z) and their derivatives and global signal were entered as covariates of no interest. Second-level analyses in the entire sample, and in the CUD and control groups separately, examined task effects related to cannabis-specific activation, using contrasts defined as cannabis > neutral and cannabis < neutral (Figure 1; Figures S1–S3 and S4–S6).

Second, whole-brain group differences were examined via a GLM (i.e., cannabis > neutral and cannabis < neutral), with age and sex as covariates, consistent with gold standards in imaging research given their profound influence on brain integrity (14, 15, 16, 17, 18). A whole-brain analysis was performed using a GLM with group as a factor (i.e., CUD vs. control). To ensure the accuracy of the results, for each contrast, we applied nonparametric threshold-free cluster enhancement (19) with 10,000 permutations, familywise error (FWE) corrected for multiple comparisons at *p* < .05. For significant clusters, we extracted the contrast beta values using a spherical region of interest (ROI) with a 5-mm radius centered on the peak voxel. The beta values were used to perform correlations between brain activity and behavioral data.

### Statistical Analyses

#### Descriptives

χ^2^ tests were performed to compare groups by sex. Mann-Whitney U tests were run to compare groups on scalar and continuous variables (i.e., IQ, substance use, mental health, craving, withdrawal, and mean valence and arousal ratings for cannabis and control images).

To analyze group differences in the mean affective valence and arousal ratings of images used in the fMRI cannabis cue-reactivity task, we first calculated the interrater reliability of all the cannabis and neutral images, respectively. We did this to determine the fit of utilizing the 2 groups’ mean scores of self-reported ratings of the images. Cronbach’s α > 0.95 indicated that ratings for all the cannabis images and all the neutral images were strongly correlated. Therefore, using participants’ mean scores for all image ratings was appropriate to create a variable for affective valence and arousal rating of cannabis and neutral images, respectively. Group differences in mean ratings were examined using nonparametric Mann-Whitney *U* tests. Cohen’s *d* effect sizes were calculated for group differences in subjective craving and arousal/valence ratings comparisons using Psychometrica’s effect size calculator for nonparametric tests.

#### Exploratory Brain-Behavior Correlations

Spearman rank correlations were performed to examine the association between differences in brain function (i.e., β coefficients from the peak activation voxels with a kernel size 5 mm from all the significant clusters), number of CUD symptoms, and variables directly relevant for cannabis cue reactivity (i.e., subjective craving), arousal ratings of cannabis images, THC-COOH:creatinine in urine, and mental health symptom scores (i.e., Cannabis Withdrawal Scale, Beck Depression Inventory, state anxiety from the State-Trait Anxiety Inventory, and the Community Assessment of Psychic Experiences positive psychotic symptoms).

In the correlations, the impact of potential confounders (i.e., cigarettes used/past month, alcohol standard drinks/past month, and hours since cannabis was used prior to testing) was accounted for via means of residualizing these from the β coefficients. Behavioral data analyses and correlations were run using SPSS version 29 (IBM Corp.).

## Results

*S*ample characteristics are summarized in Table 1. The overall sample included 108 participants (35 females and 73 males) with a median age of 25 (range: 18–56 years). Of these, 65 participants were in the moderate-to-severe CUD group and 43 in the control group.

**Table 1:** Sample Characteristics

Variable	CUD, *n* = 65	Control, *n* = 43	CUD vs. Control	
			*U*/χ^2^	*p*
Sex, Female	19	16	0.752	.386
Age, Years	27.0 (7.9)	27.8 (9.4)	1407	.955
Education, Years	15.3 (2.8)	15.6 (3.8)	1444	.469
IQ, WASI-II	106.9 (9.7)	109.0 (13.2)	1323	.428
Depression, BDI-II	11.4 (8.4)	6.3 (8.0)	708	<.001∗∗∗
State Anxiety, STAI-Y	32.7 (8.9)	29.7 (8.2)	1095	.074
Stress, PSS	15.9 (7.6)	13.4 (6.9)	1136	.159
Psychotic-Like Symptoms, CAPE				
Positive	38.7 (12.1)	30.5 (9.3)	772.5	<.001∗∗∗
Negative	40.0 (14.2)	31.9 (12.8)	931	.009∗∗
Depressive	23.0 (8.7)	18.7 (7.9)	946.5	.012∗
Alcohol				
Days/month	6 (6.8)	2.8 (4.3)	885.5	.002∗
Drinks/month	29.8 (46.0)	10.9 (18.8)	854	<.001∗∗∗
AUDIT	6.7 (4.4)	2.9 (2.7)	607	<.001∗∗∗
Nicotine				
Days/month	13.1 (13.7)	–	–	–
Cigarettes/month	64.4 (126.8)	–	–	–
FTND	1.1 (1.7)	–	–	–
Cannabis				
CUD symptoms	7.1 (1.0)	–	–	–
Days/past month	25.6 (5.2)	–	–	–
Grams/past month	26.6 (20.7)	–	–	–
Age at first try, years	16.7 (2.8)	–	–	–
Age of regular use, years	18.8 (3.5)	–	–	–
Duration, years	7.9 (7.2)	–	–	–
Lifetime exposure, grams	2295.6 (3448.4)	–	–	–
Abstinence, hours	20.9 (11.9)	–	–	–
Withdrawal, CWS	32.9 (27.5)	–	–	–
THC-COOH, urine	229.3 (236.1)	0.0 (0.0)	21	<.001∗∗∗
Craving, VAS pre-fMRI task	3.6 (2.6)	1.0 (0.0)	441	<.001∗∗∗
Craving, VAS post-fMRI task	4.6 (2.7)	1.0 (0.2)	219.5	<.001∗∗∗
COVID Stress Subscales				
Danger	4.0 (5.4)	3.4 (3.1)	720	.715
Socioeconomic	0.8 (2.6)	1.0 (2.5)	754	.321
Xenophobia	0.3 (1.0)	0.9 (2.0)	743.5	.351
Contamination	2.0 (3.7)	2.3 (3.1)	764.5	.381
Traumatic stress	1.0 (3.3)	0.2 (0.8)	596.5	.133
Checking	2.6 (3.7)	1.8 (2.2)	637	.593
VAS Rating, Arousal				
Cannabis	4.4 (2.1)	1.4 (1.0)	241.5	<.001∗∗∗
Neutral	2.7 (1.9)	2.1 (1.7)	945	.037∗
Cannabis minus neutral	1.7 (2.3)	−0.7 (1.6)	421.5	<.001∗∗∗
VAS Rating, Valence				
Cannabis	6.3 (1.3)	4.2 (1.3)	312	<.001∗∗∗
Neutral	4.2 (1.6)	4.4 (1.7)	1328.5	.524
Cannabis minus neutral	2.1 (2.4)	−0.14 (2.2)	536	<.001∗∗∗

Values are presented as mean (SD) or *n*.

∗*p* < .05, ∗∗*p* < .01, ∗∗∗*p* < .001.

AUDIT, Alcohol Use Disorders Identification Test; BDI-II, Beck Depression Inventory, Second Edition; CAPE, Community Assessment of Psychiatric Experiences; CUD, cannabis use disorder; CWS, Cannabis Withdrawal Scale; fMRI, functional magnetic resonance imaging; FTND, Fagerström Test for Nicotine Dependence; PSS, Perceived Stress Scale; STAI-Y, State-Trait Anxiety Inventory, Form Y; THC, Δ^9^-tetrahydrocannabinol; VAS, visual analog scale; WASI-II, Wechsler Abbreviated Scale of Intelligence, Second Edition.

### Sample Sociodemographic, Mental Health, and Alcohol/Nicotine Use Characteristics

Groups did not differ significantly in sex, age, IQ, state anxiety, perceived stress, or COVID-19–related stress. Compared with control participants, people with CUD had significantly higher depressive symptoms; positive, negative, and depressive psychotic-like symptoms; and greater past month alcohol exposure. Alcohol-related problems (Alcohol Use Disorders Identification Test) were significantly higher in the CUD group. Nicotine dependence (Fagerström Test for Nicotine Dependence score ≥ 3) was reported by 10 participants with CUD, while no control participants reported using nicotine.

### Cannabis Consumption and Related Problems

Participants in the CUD group endorsed a median of 7 CUD symptoms (range 4–11 symptoms), with 73.4% of them experiencing a severe CUD and the remainder experiencing a moderate CUD. Mean cannabis consumption in the CUD group was 26.6 g over 30 days in the past month, or approximately 0.7 g daily. Mean cumulative lifetime use was 898 g; the mean age of first cannabis use was 16 years, with at least monthly use commencing at a mean of 18 years and a mean duration of regular use of 7.9 years. Participants in the CUD group abstained from cannabis for a mean of 20.9 hours before testing. Cannabis withdrawal symptoms measured on the Cannabis Withdrawal Scale (CWS) had a mean of 32.9 of a possible score of 190 and ranged from no withdrawal to moderate withdrawal. Corroborating self-reported cannabis use, levels of THC-COOH:creatinine in urine were detected among participants in the CUD group but not in the control group.

### Subjective Craving Pre to Post Cannabis Cue-Reactivity fMRI Task and Arousal/Valence Ratings of Images

Subjective craving examined pre and post the cannabis cue-reactivity fMRI task was significantly higher in participants with CUD compared with control participants (*d* = 1.39, *p* < .001 and *d* = 2.00, *p* < .001, respectively). In addition, the CUD group exhibited a greater increase between time points (*d* = 0.91, *p* < .001).

Compared with control participants, individuals with a CUD rated cannabis images as triggering greater arousal levels and as having positive valence instead of neutral valence, with strong effect sizes (*d* = 1.88 and *d* = 1.65, respectively). For neutral images, arousal ratings were significantly higher in the CUD compared with the control group, with a small effect size (*d* = 0.41). Neutral image valence ratings did not differ significantly between groups. Compared with control participants, people with CUD had significantly greater arousal and valence ratings of cannabis (minus) neutral images (*d* = 1.35, *p* < .001 and *d* = 1.10, *p* < .001, respectively).

### Group Differences in Brain Activity During the Cannabis Cue-Reactivity fMRI Task

**Figure 2:**
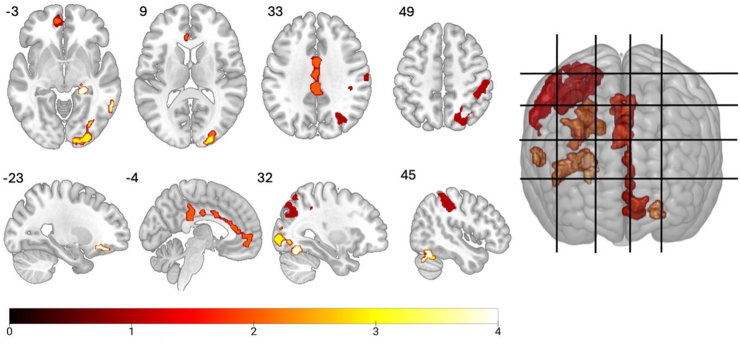
Overview of significantly greater brain activity (p < .05, familywise error corrected in axial, sagittal, and 3-dimensional views) in participants with a moderate-to-severe cannabis use disorder compared with control participants while viewing cannabis > neutral pictures. The color bar represents the *t* values for threshold-free cluster enhancement. These functional maps are overlaid on a Montreal Neurological Institute space template image. Gridlines represent the displayed slices.

Compared with control participants, participants with a moderate-to-severe CUD showed greater BOLD activity in a range of areas (*p* < .05, FWE corrected) while viewing cannabis versus neutral images (Figure 2 and Table 2). Greater activity emerged in visual/attentional areas (i.e., superior/middle occipital and fusiform gyri and calcarine sulcus); PFC regions (i.e., medial OFC, ACC); temporal regions (i.e., hippocampus, middle temporal gyrus); as well as the posterior cingulate, parietal cortices (e.g., inferior parietal, postcentral, and supramarginal gyri), and cerebellum.

**Table 2:** Group Differences in Neural Activations Associated With Cannabis Cue Reactivity (Cannabis > Neutral Images, *p* < .05, FWE Corrected)

Cluster	Peak					MNI Coordinates			Brain Regions	
k	*p*_FWE_	*p*_FDR_	TFCE	*z*	*p*_uncorrected_	x	y	z		
1092	<.05∗	<.05	897.5	3.24	<.01	−4	−30	32	Cingulate gyrus	Posterior, left
	<.05∗	<.05	759.41	3.35	<.001	−2	−12	32		Middle, left
	<.05∗	<.05	755.97	3.09	<.01	4	8	28		Anterior, right
1686	<.05∗	<.05	886	3.35	<.001	36	−44	42	Inferior parietal gyrus	Right
	<.05∗	<.05∗	855.62	3.35	<.001∗∗∗	46	−30	46	Postcentral gyrus	Right
	<.05∗	<.05∗	849.43	3.24	<.01∗∗	44	−32	38	Supramarginal gyrus	Right
449	<.05∗	<.05∗	871.21	3.35	<.001∗∗∗	44	−60	−22	Cerebellum	Crus 1, right
	<.05∗	<.05∗	767.39	3.24	<.01∗∗	48	−54	−26		
	<.05∗	<.05∗	830.81	3.24	<.01∗∗	28	−66	−14	Fusiform gyrus	Right
579	<.05∗	<.05∗	782.06	3.54	<.001∗∗∗	18	−96	−2	Calcarine sulcus	Right
	<.05∗	<.05∗	767.07	3.35	<.001∗∗∗	24	−100	6	Occipital gyrus	Superior, right
	<.05∗	<.05∗	758.42	3.35	<.001∗∗∗	24	−88	6		Middle, right
59	<.05∗	<.05∗	704.6	3.54	<.001∗∗∗	−24	38	−16	OFC, frontal gyrus, orbital	Inferior, left
	<.05∗	<.05∗	686.61	3.35	<.001∗∗∗	−24	28	−12		Middle, left
55	<.05∗	<.05∗	700.89	3.24	<.01∗∗	24	−34	−4	Hippocampus	Right
27	<.05∗	<.05∗	667.15	3.24	<.01∗∗	26	−22	−10		
67	<.05∗	<.05∗	679.89	3.04	<.01∗∗	62	−52	−6	Middle temporal gyrus	Right

All coordinates are presented in MNI space. Brain regions identified using the Automated Anatomical Labeling atlas. Age and sex were included as covariates in the general linear model. *p* < .05, FWE corrected.

∗*p* < .05, ∗∗*p* <.01, ∗∗∗*p* < .001.

FDR, false discovery rate; FWE, familywise error; MNI, Montreal Neurological Institute; OFC, orbitofrontal cortex; TFCE, threshold-free cluster enhancement.

### Brain-Behavior Correlations

Table 3 shows correlations between brain activity and behavioral variables, accounting for cigarettes used/past month, alcohol standard drinks/past month, and hours since cannabis was last used prior to testing. In the CUD group, there were positive correlations between greater superior occipital cortex activity and arousal ratings of cannabis (minus) neutral images (VAS scores; rho = 0.33, *p* < .05) and withdrawal (CWS scores; rho = 0.32, *p* < .05). THC-COOH:creatinine levels negatively correlated with activity of the ACC and inferior parietal cortex (rho = −0.28, *p* < .05 and rho = −0.30, *p* < .05, respectively). These correlations were still significant after outlier removal. Additional correlations did not survive outlier removal or were nonsignificant.

**Table 3:** Brain-Behavior Correlations in Participants With CUD

	Peak Number		Craving			Arousal			CUD Symptoms			THC-COOH			Withdrawal			Depression			State Anxiety			Positive Psychotic Symptoms		
			Rho	*n*	*p*	Rho	*n*	*p*	Rho	*n*	*p*	Rho	*n*	*p*	Rho	*n*	*p*	Rho	*n*	*p*	Rho	*n*	*p*	Rho	*n*	*p*
Cingulate Gyrus	Posterior, left	_	−0.03	51	.83	0.21	49	.14	−0.14	51	.34	−0.23	51	.11	−0.10	50	.47	0.09	50	.56	0.07	50	.65	0.16	49	.29
	Middle, left	_	−0.16	51	.27	0.21	49	.15	−0.04	51	.78	−0.13	51	.37	0.07	50	.62	−0.07	50	.63	0.07	50	.61	0.03	49	.83
	Anterior, right	_	−0.03	51	.83	−0.01	49	.96	−0.08	51	.57	−0.28	51	<.05∗^,^^a^	0.07	50	.66	0.06	50	.68	0.10	50	.47	0.16	49	.28
Inferior Parietal Gyrus	Right	_	−0.22	51	.12	−0.13	49	.39	−0.19	51	.18	−0.30	51	<.05∗^,^^a^	0.08	50	.60	0.08	50	.61	0.15	50	.28	0.14	49	.33
Postcentral Gyrus	Right	_	−0.14	51	.33	−0.12	49	.41	−0.15	51	.31	−0.30	51	<.05∗	0.01	50	.95	0.00	50	.99	0.11	50	.45	0.15	49	.30
Supramarginal Gyrus	Right	_	−0.22	51	.12	−0.07	49	.63	−0.23	51	.11	−0.28	51	<.05∗	−0.12	50	.42	−0.02	50	.87	0.03	50	.82	−0.04	49	.77
Cerebellum	Crus 1, right	1	0.06	51	.70	−0.06	49	.69	0.17	51	.23	−0.07	51	.62	0.34	50	<.05∗	0.04	50	.78	−0.03	50	.83	−0.01	49	.94
		2	−0.04	51	.78	−0.08	49	.56	0.22	51	.13	−0.07	51	.63	0.23	50	.11	0.06	50	.69	0.06	50	.69	−0.11	49	.45
Fusiform Gyrus	Right	_	0.06	51	.65	−0.13	49	.39	−0.10	51	.48	0.10	51	.47	0.28	50	.05	−0.06	50	.68	−0.18	50	.21	0.06	49	.66
Calcarine Sulcus	Right	_	0.18	51	.21	0.00	49	.99	−0.08	51	.60	0.05	51	.75	0.28	50	.05	0.29	50	<.05∗	0.16	50	.28	0.18	49	.21
Occipital Gyrus	Superior, right	_	0.13	51	.38	0.33	49	<.05∗^,^^a^	−0.09	51	.52	0.03	51	.83	0.32	50	<.05∗^,^^a^	0.30	50	<.05∗	0.09	50	.51	0.09	49	.54
	Middle, right	_	0.09	51	.55	0.19	49	.18	0.11	51	.44	0.24	51	.09	0.33	50	<.05∗	0.34	50	<.05∗	0.12	50	.42	0.08	49	.60
OFC, Frontal Gyrus, Orbital	Inferior, left	_	0.23	51	.11	0.17	49	.25	−0.07	51	.62	−0.10	51	.47	0.14	50	.32	0.14	50	.32	0.25	50	.08	0.17	49	.25
	Middle, left	_	0.10	51	.47	0.09	49	.54	−0.12	51	.42	−0.24	51	.09	−0.13	50	.38	−0.16	50	.26	0.00	50	.98	0.15	49	.32
Hippocampus	Right	1	−0.01	51	.96	0.07	49	.65	0.10	51	.49	−0.05	51	.73	0.07	50	.65	−0.01	50	.97	−0.08	50	.59	0.06	49	.69
		2	−0.12	51	.41	0.11	49	.46	−0.04	51	.79	0.06	51	.68	0.12	50	.41	0.08	50	.59	−0.03	50	.81	−0.14	49	.36
Middle Temporal Gyrus	Right	_	−0.03	51	.82	0.16	49	.29	−0.19	51	.19	−0.25	51	.08	−0.02	50	.90	0.02	50	.88	0.04	50	.79	0.09	49	.52

Blood oxygen level–dependent activations preresidualized to control for the effects of number of standard alcohol drinks consumed in the past month, cigarettes consumed in the past month, and abstinence (hours since last cannabis use). Arousal indicates arousal ratings for cannabis > neutral images; craving indicates subjective craving following the cannabis cue-reactivity functional magnetic resonance imaging task; depression indicates Beck Depression Inventory total score; positive psychotic symptoms indicates a subscale of the Community Assessment of Psychic Experience; state anxiety indicates state subscale of the State-Trait Anxiety Inventory; withdrawal indicates Cannabis Withdrawal Scale.

∗*p* < .05.

CUD, cannabis use disorder; OFC, orbitofrontal cortex; THC, Δ^9^-tetrahydrocannabinol.

a Correlation was robust to outlier removal.

## Discussion

To our knowledge, this is the largest fMRI study to examine brain function during cannabis cue reactivity, for the first time in people with moderate-to-severe CUD who tried to cut down or quit and comprehensively characterized for cannabis use levels compared with control participants. We found that compared with control participants, during cannabis cue reactivity, individuals with CUD exhibited greater activity in the ACC and inferior parietal cortex, which correlated with urinary THC-COOH levels. The CUD group also showed greater brain activity in the visual regions, which correlated with higher arousal ratings of cannabis images and withdrawal scores. Furthermore, compared with control participants, participants with CUD showed greater activity in the OFC, occipito-temporal regions (i.e., hippocampus, middle temporal gyrus, fusiform gyrus, and calcarine sulcus), posterior cingulate, parietal cortices (e.g., inferior parietal, postcentral and supramarginal gyri), and cerebellum. Interestingly, we reported a lateralization of activations—left for the more anterior brain finding and right for the posterior regions. No bilateral findings were noted for the same brain regions. In a previous literature review (10), significant lateralization did not emerge as a key feature of CUD, even though lateralization effects were noted for posterior occipital regions in previous work (9,20, 21, 22). Whether lateralization of anterior and posterior brain pathways characterizes CUD needs to be corroborated by future work.

Compared with control participants, people with CUD exhibited greater PFC activity during cannabis cue reactivity, encompassing the OFC and the ACC—both regions implicated in regulating responses to salient drug-related stimuli (12). This finding was consistent with our hypothesis, fMRI evidence on cannabis cue-reactivity tasks with people who use cannabis (13) and other substances (23), and neuroscientific theories of addiction (7). The OFC regulates limbic-striatal regions implicated in reward processing, motivation, and effortful control (12). Thus, greater OFC cue reactivity may reflect an increased attribution of salience and reward expectancy to cannabis cues (24). Meanwhile, the ACC is implicated in supporting awareness of negative emotional states (7). Therefore, the correlation between greater ACC activity and lower THC metabolites in urine may indicate that participants with lower THC metabolites experience greater reactivity to cannabis cues, prompting a need to use cannabis.

The most robust aspect of cannabis cue reactivity (to date) is arguably visual processing, and participants with CUD showed stronger activity in occipital areas implicated in the processing of higher-order visual stimuli (25), consistent with previous studies on cannabis (10) and other substances (26). We also found greater cerebellar activity in people with CUD while viewing cannabis versus neutral images, consistent with previous evidence from people who use cannabis (10) and other substances [e.g., alcohol (27)]. Notably, the cerebellum is a core component of the addiction neurocircuitry (7) and exhibits a high density of cannabinoid CB_1_ receptors, where THC exerts its psychoactive effects (28). The cerebellum is also implicated in drug-associated cue memory, and cue-elicited cerebellar activity may reflect an anticipatory response to cannabis cues (28).

The greater activation of the hippocampus and medial temporal areas in participants with CUD compared with control participants is consistent with existing fMRI evidence (10). Hippocampal and temporal regions are implicated in stress and verbal/visuospatial learning and memory processes (29) known to be altered in this population (30). Thus, cannabis cue reactivity might have triggered greater activation of stress and cue-related memory circuits (29).

The activity of parietal regions was also greater in the CUD group compared with control participants (i.e., inferior parietal, postcentral, supramarginal gyri). The reported alteration in parietal activity is consistent with fMRI cannabis cue-reactivity evidence from people who use cannabis and other substances (10,31,32) and prominent neuroscientific theories of addiction (7). Interestingly, the postcentral gyrus, reportedly enhanced in cannabis users, is an integral part of the sensorimotor network implicated in the integration of sensory stimuli with emotions and memories (33,34), while the supramarginal gyrus is implicated in episodic memory (35) and is also altered in people who use cannabis (36). Therefore, exposure to cannabis cues in CUD may aid recollection of previous cannabis use experiences and their context (e.g., associated emotions, sensory experiences, time, place) and trigger craving and disinhibition of cannabis use behavior.

In contrast with the hypothesis, there was no group difference in brain activity in the striatum, a key hub for reward processing in the addiction neurocircuitry (7). However, its function is difficult to measure with neuroimaging modalities/techniques due to its high concentration of iron (37). It is possible that ROI data acquisition and analysis approaches (instead of the whole-brain exploratory approaches used in this work) are required to measure changes in brain function of the striatum with precision.

Subjective craving significantly increased pre to post the cannabis cue-reactivity fMRI task, and the CUD group (not the control group) reported greater subjective valence/arousal in relation to cannabis versus neutral images, suggesting that the stimuli and the cue-exposure task might have triggered increases in craving. However, subjective craving did not correlate with brain function, which is consistent with some studies but not others (10). The relationship between cue-elicited brain function and subjective craving may be modulated by other factors, e.g., perceived availability of cannabis use postpresentation of cannabis cues (38), and varying levels of CUD symptoms, which were unmeasured. It is possible that interindividual variability in craving was too low to capture its association with brain function (i.e., a narrow range of subjective craving), and meta-regressions of the literature may be needed to explore this association.

Overall, the regions involved in cannabis cue reactivity in CUD are critical components of 3 distinct systems previously implicated in craving (39): the visual system to process the cannabis cues (e.g., occipital regions), the motivational/inhibitory systems implicated in top-down control (e.g., OFC, ACC, and parietal regions), and the subcortical temporolimbic system implicated in stress and cue-related memory activation (e.g., hippocampus, cerebellum). Functional connectivity analyses are required to examine the functional communication between these systems.

The findings from this study need to be considered in light of several methodological limitations. First, the cross-sectional design prevents understanding of whether group differences predated CUD (40) or changed over time as people progressed to greater or lower CUD severity, relapse, or following prolonged abstinence. Longitudinal studies are required to test this notion. Second, we lacked objective proxies for craving (e.g., skin conductance) and stress (e.g., cortisol), which should be integrated into future studies to precisely map the multimodal mechanisms of cannabis cue reactivity in CUD. Because the study was restricted to people with moderate-to-severe CUD and a history of attempting to quit or cut down due to our focus on cannabis users who experience difficulties with use, the findings may not generalize to other groups of cannabis users (e.g., mild CUD or no CUD). Third, importantly, the study was conducted during the COVID pandemic. While we cannot rule out the possibility that COVID could have affected cannabis use behavior as previously suggested (41,42), in our sample, we did not find any significant correlations between any of the COVID-stress subscale scores (i.e., socioeconomic consequences, xenophobia, danger fears, contamination fears, traumatic stress, compulsive checking, and reassurance seeking) and cannabis quantity and use days in the past month (Table S7). Furthermore, the CUD and control groups did not differ in any of the COVID-stress scale subscales, suggesting that the 2 groups might have been affected in a comparable fashion.

Fourth, the cannabis cue-reactivity fMRI task used herein robustly induced craving and triggered cue reactivity (20) and was robust in that cannabis and neutral stimuli were well matched according to various characteristics (e.g., complexity, size, brightness, color, resolution) (43). However, specific/personalized cues may be required to trigger cue-induced craving for a more naturalistic design that generalizes to individual experiences outside the scanner (22). Finally, the study lacked control images with rewarding properties (e.g., monetary) that also recruit brain reward pathways altered in people who use cannabis (44). The findings need to be replicated using additional rewarding stimuli to disambiguate whether cannabis cue reactivity was specific to cannabis images or applies to rewarding images generally.

Reactivity to cues has been associated with relapse in other substance use disorders (SUDs) (45). Our findings provide a preliminary foundation for informing treatment targets for individuals with more severe CUDs who are vulnerable to relapse (46). Brain-based interventions that target brain functional alterations specific to the individual during cannabis cue reactivity—such as fMRI-based neurofeedback and transcranial magnetic stimulation (47)—could prove useful to reduce individual-specific reactivity to cannabis cues. Therefore, such treatments could inform the development of personalized interventions to prevent relapse and support long-term recovery in individuals who experience CUDs. Such brain-based interventions could be combined with therapies that strengthen top-down executive control or reduce bottom-up automatic responses to cues [e.g., cognitive bias modification (48), mindfulness-based relapse prevention (49)]. Our findings also support the investigation of interventions that subjectively devalue cannabis by reducing its expected reward while increasing the relative valuations of nondrug rewards.

### Conclusions

The findings suggest that cannabis cue reactivity in individuals with moderate-to-severe CUD who tried to cut down or quit is associated with greater activity of the prefrontal, temporal, and parietal regions of the addiction neurocircuitry (12). It is also associated with additional activity in the occipital regions implicated in higher-order visual/attention processing and with cannabis withdrawal, depressive symptom scores, and arousal elicited by the images administered during the cannabis cue-reactivity fMRI task. The neurobiology of cannabis cue reactivity in CUD may affect fronto-temporo-parietal neurocircuitry overlapping with other SUDs [i.e., alcohol, nicotine, opioids, cocaine (45)] and may (in part) be consistent with prominent neuroscientific theories of addiction (12). Our findings have implications for the development of psychological and brain-based interventions that aim to target the function of these regions to decrease craving, cannabis dosage, and the severity of CUD.

## Supplementary Material

Supplemental Data, Figures S1–S3, and Tables S1–S7

Key Resources Table
