# Supplementary material for: The Neurocircuitry of Cannabis Cue Reactivity in Cannabis Use Disorder: A Functional Neuroimaging Study
Source: Biol Psychiatry Glob Open Sci. 2025 Oct 16;6(1):100638. doi: 10.1016/j.bpsgos.2025.100638 (PMC12720038; doi:10.1016/j.bpsgos.2025.100638)
Supplement: Supplemental Data, Figures S1–S3, and Tables S1–S7 [file mmc1.pdf]

SUPPLEMENTARY INFORMATION

The Neurocircuitry of Cannabis Cue-Reactivity in Cannabis Use Disorder: A Functional Neuroimaging Study

Lorenzetti *et al.*

Supplemental data

1. Inclusion and exclusion criteria

1.1 Inclusion criteria

All participants:

i) age between 18 and 56 years;

ii) proficiency in English; and

iii) normal or corrected-to-normal vision.

Participants with a CUD:

i) endorsed DSM-5 criteria for a moderate-to-severe CUD based on the Structured Clinical Interview for DSM-5 Research Version (SCID-5-RV),

ii) consumed cannabis daily/almost daily for at least the past 12-months, and

iii) reported at least one attempt to quit or to reduce their use in the past 24 months.

1.2 Exclusion criteria

All participants:

i) significant medical conditions, history of acquired brain injury or loss of consciousness > 5 minutes;

ii) history of psychopathology (except for depression and anxiety) ascertained by the Mini International Neuropsychiatric Interview (MINI);

iii) illicit drug and alcohol use in the 12-hours before testing determined by self-report;

iv) significant alcohol use or dependence (i.e., Alcohol Use Disorder Identification Test (AUDIT (1); score ≥ 14));

v) illicit drug use (other than cannabis in the CUD group) in the past 4-weeks or above recreational levels (i.e., > 50 lifetime episodes, or > weekly use over a 3-month period) confirmed by a comprehensive questionnaire previously used by our team (2-4);

vi) current use of prescription medication that affects the central nervous system with the exception of antidepressants, e.g., selective serotonin reuptake inhibitors (SSRI's) and serotonin-norepinephrine reuptake inhibitor (SNRI's), due to increased prevalence of depression and anxiety in CUD populations and our inclusion of these mental health disorders (5);

vii) MRI contraindications (e.g., pacemaker, pregnancy) confirmed by a screener from the testing facility: https://www.monash.edu/__data/assets/pdf_file/0004/972022/pqms3-mbi-frm-c001-mri-screening-information-form.pdf; and

viii) IQ scores < 80 determined by the vocabulary and matrix reasoning subtests of the Weschler Abbreviated Standardised Intelligence-II (6).

2. Process of participants' selection against inclusion and exclusion criteria

A total of 9,030 people completed an the online screening survey against study' inclusion and exclusion criteria. The measures used to address this included (i) demographics; (ii) MRI screening; (iii) previous research participation; (iv) handedness; (v) past and current cannabis use related questions; (vi) alcohol use history; (vii) substance use history; (viii) mental health screening; (ix) depressive symptoms; (x) COVID-19 related stress and; (xi) attempts to quit cannabis. Of these, 7,980 did not meet study eligibility or did not fully complete the screener. About ~1,050 people who were potentially eligible in the study underwent a phone follow up to confirm eligibility. Any queries about participants' eligibility were resolved via discussion with the study CI and the research team, before confirming inclusion or exclusion.

A total of 117 participants aged 18-56 years (39 female) were included for face-to-face behavioural/MRI testing. This is where eligibility criteria were confirmed. The sample included

one male participant aged 19 who has been using cannabis for 11 months (i.e., less than 12 month cannabis use history) who met eight DSM-5 criteria for a CUD (i.e., severe) and used daily. Nine participants were excluded after face-to-face testing when it emerged they met exclusion criteria.

2.1 Reimbursement

Participants were reimbursed via Coles/Myers vouchers of $100 for controls and $150 for the CUD group (additional reimbursement was to compensate for further research activities part of the broader study).

3. Assessment of Sociodemographic, substance use and mental health data

Questionnaires measured sociodemographic variables, including age and sex. Metrics of substance use and related problems and mental health.

3.1 Metrics of cannabis exposure and related problems

The *Structured Clinical Interview for DSM-5 Research Version* (SCID-5-RV) was administered to determine the severity of CUD for inclusion and the number of symptom scores endorsed (7). The *Cannabis Withdrawal Scale* (CWS) was administered to measure withdrawal symptoms (8); while 9-point visual analogue scales (VAS) were given to rate participant's ratings of *arousal and affective valence* of all the cannabis and neutral images that were viewed during the fMRI cannabis cue-reactivity task. Arousal was rated from 1 representing "*calm*" to 9 "*excited*"; and affective valence was rated on a scale from 1 "*unpleasant*", to 9 "*pleasant*" with a rating of 5 representing "*neutral*". A 10-point VAS scale was also administered to measure cannabis cravings immediately before and after the fMRI cannabis cue-reactivity task,

with the item *"how much do you feel like smoking cannabis right now?",* and answers ranging from '1' indicating *"not at all"* to '10' "*extremely"*.

A *semi-structured interview* used in previous studies was administered to measure lifetime cannabis use history, from which we extracted key cannabis exposure parameters accounting for periods of abstinence (3, 4, 9). They were: age when cannabis was first tried, age of regular cannabis use (i.e., defined as ≥ 2 days/month), duration of regular cannabis use (i.e., defined as at a rate of ≥ 2 days/month), and cumulative lifetime exposure in grams. The *Timeline Follow-Back* was administered to measure past month: days of use, dosage in grams, hours since cannabis was last use (10).

Presence of cannabis use was corroborated via presence of *11-nor-9-carboxy-Δ^9-tetrahydrocannabinol:creatinine* (THC-COOH:creatinine) in urine, which measure THC concentration combined with creatinine which controls for the variation in THC due to varying hydration status of participants. It was confirmed by toxicology analyses conducted by Drugs and Toxicology Group, Centre for Forensic Science, University of Technology Sydney (led by Professor Shanlin Fu).

3.2 Metrics of exposure to other substances and substance use-related problems

The *Timeline Follow-Back* was administered to extract days of use and dosage for alcohol (i.e., standard drinks) and nicotine use over the past 30 days (10). Alcohol and nicotine dependence were assessed via the *AUDIT* and the *Fagerström Test for Nicotine Dependence* (FTND; (11)).

3.3 Mental health symptom scores

The *Beck Depression Inventory* – Second Edition (12), the *State-Trait Anxiety Index – Y Form* (13), and the *Perceived Stress Scale* (14) were administered to measure depression, anxiety and perceived stress symptoms. The Community Assessment of Psychic Experiences (CAPE) was

given to meaure positive and negative psychositc symptoms, and depressive symptoms (15). The *COVID-19 Stress Scales* were administered to measure COVID related stress in relation to: danger, socioeconomic consequences, xenophobia, contamination, traumatic stress, and compulsive checking (16).

4. MRI data acquisition

The neuroimaging data acquisition is outlined in detail in the following section and Supplemental Figure 1.

4.1 Structural MRI

MRI images were acquired on a Siemens Skyra 3 Tesla scanner using a 32-channel head coil at the Monash Biomedical Imaging facility. Brain images were acquired in coronal view, from anterior to posterior. Structural MRI data was acquired using T1-weighted MPRAGE scan with the following acquisition parameters were: TE = 2.07ms, TR = 2300ms, flip angle = 9°, 192 slices without gap, FOV 256x256mm, 1x1x1 mm voxels, and total acquisition time of ~5 minutes and 20 seconds.

4.2 fMRI cannabis cue-reactivity task

fMRI data during the cannabis cue-reactivity task was acquired using T2* weighted EPI scans. Acquisition parameters were: TR = 2240ms, TE = 30ms, flip angle = 90°, field of view = 192mm, matrix = 64, voxel size 3 x 3 x 3mm^3, 40 slices, with 227 total volumes. The task total acquisition time was ~8 minutes and 37 seconds.

4.1. Task description

An event-related cannabis cue-reactivity fMRI task adapted from previous experiments from our extended team (17) was used to measure brain activity while participants passively watched

30 cannabis and 30 neutral non-cannabis images used as cues of comparable resolution, type of activity, size, brightness, and luminance (Supplemental Figure 1).

The images showed cannabis-related paraphernalia and use behaviours, and neutral images depicted stationary items or cooking utensils. Each image was presented for 4 seconds preceded by a fixation-cross that lasted on average 4 seconds, jittered between 2 and 6 seconds. The cannabis and neutral images were presented in the same semi-random order (max three images of the same category in a row) for each participant.

Participants were instructed to pay close attention to the images "*In this task you will see pictures on the screen. Please try to keep your head still. Your task is to look at these pictures closely and as attentively as you can. The task will take about 10 minutes. We are about to start. Are you ready?*". To ensure that participants maintained attention, their alertness was monitored via an MRI compatible camera and any occasions of sleepiness prompted a re-start of the scan. Images were presented onto a rear projection screen positioned behind the MRI scanner using E-prime3 software (Version 3.0 Build 3.0.3.80, E-Studio Build 3.0.3.82, Psychology Software Tools Inc.). Total task time was ~10 minutes. See Supplemental Tables 1 and 2 for precise image presentation order and trial timing information, respectively.

Supplemental Table 1: Order of image file presentation for task versions A and B.

Presentation order	Version A		Version B	
	Stimulus valence	Image file	Stimulus valence	Image file
1	neutral	N18.jpg	neutral	C3.jpg
2	neutral	N23.jpg	neutral	C13.jpg
3	cannabis	C16.jpg	cannabis	N21.jpg
4	cannabis	C9.jpg	cannabis	N28.jpg
5	neutral	N24.jpg	neutral	C25.jpg
6	cannabis	C4.jpg	cannabis	N8.jpg
7	neutral	N20.jpg	neutral	C7.jpg
8	neutral	N2.jpg	neutral	N4.jpg
9	cannabis	C23.jpg	cannabis	C21.jpg
10	cannabis	C28.jpg	cannabis	N19.jpg
11	cannabis	C30.jpg	cannabis	C5.jpg
12	neutral	N7.jpg	neutral	C6.jpg
13	cannabis	C10.jpg	cannabis	N17.jpg
14	cannabis	C29.jpg	cannabis	N16.jpg
15	neutral	N10.jpg	neutral	C27.jpg
16	cannabis	C22.jpg	cannabis	N5.jpg
17	cannabis	C20.jpg	cannabis	N13.jpg
18	cannabis	C18.jpg	cannabis	C26.jpg
19	neutral	N30.jpg	neutral	N3.jpg
20	neutral	N1.jpg	neutral	C12.jpg
21	neutral	N15.jpg	neutral	C19.jpg
22	cannabis	C17.jpg	cannabis	N14.jpg
23	cannabis	C14.jpg	cannabis	N27.jpg
24	neutral	N6.jpg	neutral	N29.jpg
25	neutral	N12.jpg	neutral	C24.jpg
26	cannabis	C8.jpg	cannabis	C1.jpg
27	neutral	N9.jpg	neutral	N26.jpg
28	cannabis	C11.jpg	cannabis	N25.jpg
29	neutral	N22.jpg	neutral	C15.jpg
30	neutral	N11.jpg	neutral	C2.jpg
31	cannabis	C3.jpg	cannabis	N18.jpg
32	cannabis	C13.jpg	cannabis	N23.jpg
33	neutral	N21.jpg	neutral	C16.jpg
34	neutral	N28.jpg	neutral	C9.jpg
35	cannabis	C25.jpg	cannabis	N24.jpg
36	neutral	N8.jpg	neutral	C4.jpg
37	cannabis	C7.jpg	cannabis	N20.jpg
38	neutral	N4.jpg	neutral	N2.jpg

39	cannabis	C21.jpg	cannabis	C23.jpg
40	neutral	N19.jpg	neutral	C28.jpg
41	cannabis	C5.jpg	cannabis	C30.jpg
42	cannabis	C6.jpg	cannabis	N7.jpg
43	neutral	N17.jpg	neutral	C10.jpg
44	neutral	N16.jpg	neutral	C29.jpg
45	cannabis	C27.jpg	cannabis	N10.jpg
46	neutral	N5.jpg	neutral	C22.jpg
47	neutral	N13.jpg	neutral	C20.jpg
48	cannabis	C26.jpg	cannabis	C18.jpg
49	neutral	N3.jpg	neutral	N30.jpg
50	cannabis	C12.jpg	cannabis	N1.jpg
51	cannabis	C19.jpg	cannabis	N15.jpg
52	neutral	N14.jpg	neutral	C17.jpg
53	neutral	N27.jpg	neutral	C14.jpg
54	neutral	N29.jpg	neutral	N6.jpg
55	cannabis	C24.jpg	cannabis	N12.jpg
56	cannabis	C1.jpg	cannabis	C8.jpg
57	neutral	N26.jpg	neutral	N9.jpg
58	neutral	N25.jpg	neutral	C11.jpg
59	cannabis	C15.jpg	cannabis	N22.jpg
60	cannabis	C2.jpg	cannabis	N11.jpg

N.B. participants were randomised to receive either version A or version B at roughly equal proportions (50.7% and 49.3% respectively).

Supplemental Table 2: Trial duration ordered (from lowest to highest) based on variable fixation cross duration (jitter).

Fixation cross duration	Stimulus valence	Image file	Stimulus duration	Trial duration
2097	cannabis	C19.jpg	4000	6097
2116	neutral	N11.jpg	4000	6116
2230	neutral	N14.jpg	4000	6230
2298	cannabis	C22.jpg	4000	6298
2379	cannabis	C16.jpg	4000	6379
2433	cannabis	C26.jpg	4000	6433
2468	neutral	N29.jpg	4000	6468
2526	neutral	N23.jpg	4000	6526
2615	neutral	N1.jpg	4000	6615
2676	cannabis	C21.jpg	4000	6676
2721	cannabis	C28.jpg	4000	6721
2861	neutral	N27.jpg	4000	6861
2864	cannabis	C9.jpg	4000	6864
2990	neutral	N5.jpg	4000	6990
3012	neutral	N15.jpg	4000	7012
3096	cannabis	C27.jpg	4000	7096
3199	cannabis	C29.jpg	4000	7199
3214	cannabis	C2.jpg	4000	7214
3256	neutral	N26.jpg	4000	7256
3314	neutral	N22.jpg	4000	7314
3433	neutral	N10.jpg	4000	7433
3494	cannabis	C25.jpg	4000	7494
3548	cannabis	C30.jpg	4000	7548
3604	cannabis	C4.jpg	4000	7604
3670	neutral	N16.jpg	4000	7670
3760	neutral	N19.jpg	4000	7760
3876	neutral	N12.jpg	4000	7876
3898	cannabis	C7.jpg	4000	7898
3977	cannabis	C5.jpg	4000	7977
4031	neutral	N18.jpg	4000	8031
4079	cannabis	C14.jpg	4000	8079
4156	neutral	N25.jpg	4000	8156
4165	cannabis	C20.jpg	4000	8165
4219	cannabis	C3.jpg	4000	8219
4254	neutral	N13.jpg	4000	8254
4301	cannabis	C15.jpg	4000	8301
4432	neutral	N9.jpg	4000	8432
4500	neutral	N2.jpg	4000	8500
4594	cannabis	C11.jpg	4000	8594
4665	cannabis	C8.jpg	4000	8665

4675	neutral	N21.jpg	4000	8675
4768	neutral	N17.jpg	4000	8768
4823	cannabis	C1.jpg	4000	8823
4945	cannabis	C13.jpg	4000	8945
5043	neutral	N20.jpg	4000	9043
5068	cannabis	C10.jpg	4000	9068
5172	cannabis	C23.jpg	4000	9172
5220	neutral	N3.jpg	4000	9220
5263	neutral	N4.jpg	4000	9263
5311	cannabis	C12.jpg	4000	9311
5447	cannabis	C24.jpg	4000	9447
5465	neutral	N6.jpg	4000	9465
5532	neutral	N30.jpg	4000	9532
5627	cannabis	C17.jpg	4000	9627
5659	neutral	N28.jpg	4000	9659
5766	cannabis	C18.jpg	4000	9766
5827	neutral	N8.jpg	4000	9827
5831	cannabis	C6.jpg	4000	9831
5880	neutral	N7.jpg	4000	9880
5985	neutral	N24.jpg	4000	9985

N.B. All durations are in milliseconds (ms)

4.3 MRI data pre-processing

fMRI data pre-processing steps were completed via fMRIPrep (version 20.2.3) (18): distortion correction, head motion correction, slice timing, co-registration with T1-weights images normalised to standard space (i.e., Montreal Neurological Institute [MNI] space), and smoothing with 6 mm Gaussian kernel. Image quality was assessed (i.e., motion, signal-noise ratio, artifacts) against conservative criteria validated for resting-state fMRI via review of Framewise Displacement (FD; indicator of motion, including data < 0.5 mm cutoff) (19). No participants were excluded after fMRI quality checks.

5. Overview of participants excluded from analyses after testing

Nine participants (6 with a CUD and 3 controls) were excluded due to subsequently meeting exclusion criteria when face-to-face testing. The people with a CUD who were excluded

were: a female aged 26 years with an IQ score < 80 (i.e., FSIQ-2 = 61), 1 male aged 22 years endorsing a neurological disorder (i.e., history of seizures), 3 participants (male aged 31, male aged 32, female aged 25) with incidental findings determined by a neurologist that conducted comprehensive checks of MRI images, and a male aged 31 who used synthetic cannabis. The three controls excluded reported > 50 lifetime occasions of cannabis use. They were: i) a female aged 32 years, who reported ~4000 lifetime occasions over 13.5 years, ii) a female aged 51 years, who reported 176 lifetime occasions since the age of 18, and 1 male aged 35 years, who reported 420 lifetime occasions in 14-months and endorsed a history of a diagnosed psychiatric condition in adolescence.

6. Occasional illicit substance use

As outlined in Supplemental Table 3, 20 participants used illicit substances other than cannabis, of which 2 were controls. They used substances other than cannabis over the 4-week period prior to scanning, on a median of 1 occasion (range 1 – 6 occasions), for a median of 229 hours before testing (range 16 – 648 hours).

Supplemental Table 3. Overview of participants' occasional exposure to illicit substances within 4 weeks from testing

Substance	Group	Age	Sex	Hours before testing	Dosage
Amyl Nitrate	Control	20	male	48	1 dose
Cocaine	CUD	22	male	624	1 gram
	CUD	19	female	552	1 gram
	CUD	29	male	38	2.5 gram
	CUD	31	male	392	0.1 gram
	CUD	27	male	432	0.3 gram
	CUD	21	male	229	0.8 gram
	CUD	24	male	139	50 mg
Dexamphetamine	CUD	35	male	29	20 mg
Ketamine	CUD	21	male	240	0.5 gram
MDMA	CUD	39	male	108	1 dose
	CUD	21	male	368	0.5 gram
	Control	29	male	60	0.1 gram
	CUD	28	female	16	0.15 gram
	CUD	25	male	546	0.1 gram
Mescaline	CUD	18	male	648	1 dose
Modafinil	Control	29	male	432	50 mg
	CUD	29	male	96	0.2 gram
Mushrooms	CUD	21	male	638	2 gram
	CUD	22	male	148	1.8 gram
	CUD	18	male	648	2 gram
	CUD	22	male	504	1 gram
	CUD	26	male	137	1 gram
Valium	CUD	21	female	40	5 mg
Vyvanse	CUD	21	female	28	45 mg
Xanax	CUD	18	female	360	1 mg
	CUD	19	male	159	_

Abbreviations: MDMA, methylenedioxy-methylamphetamine; _, missing; mg, milligrams.

7. Brain activity in the whole sample during cannabis cue-reactivity fMRI task

We examined brain function in the whole sample during cannabis cue-reactivity fMRI task as 'sanity check' to ensure the task recruited the expected neurocircuitry for the contrasts cannabis > neutral and neutral > cannabis.

In the whole sample, there was greater brain activity while participants viewed *cannabis* > *neutral* images (Supplemental Figure 1 and Supplemental Table 4) in a range of frontal, temporo-parietal and visual cortical regions brain regions: superior frontal gyrus medial/dorsolateral, middle temporal/occipital gyrus, anterior cingulate & paracingulate gyri, angular gyrus, middle temporal gyrus, calcarine fissure and supramarginal gyrus. Meanwhile, for the contrast *neutral* > *cannabis*, the whole sample showed greater activity in additional areas: inferior/middle occipital gyrus, precentral gyrus, inferior frontal gyrus (i.e., pars triangularis and pars opercularis), supplementary motor area, and superior medial OFC.

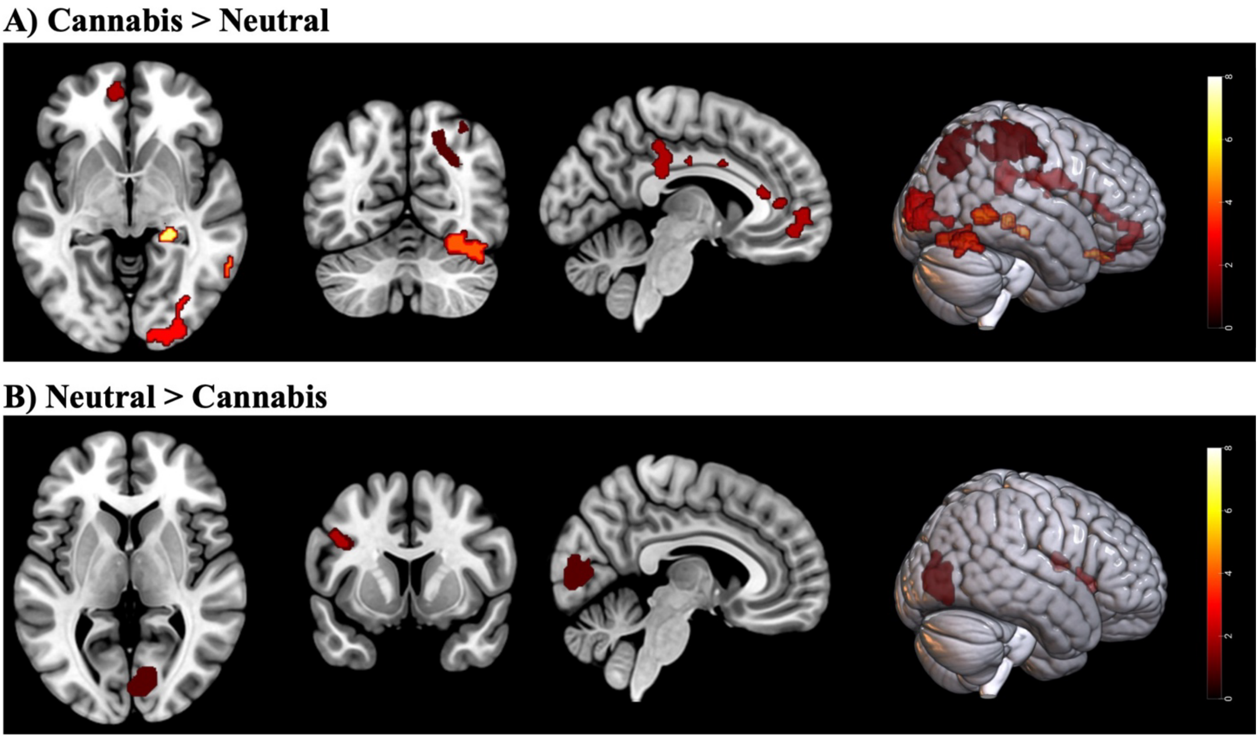

Supplemental Figure 1. Brain regions of differences for the whole sample during the contrasts A) cannabis > neutral, and B) neutral > cannabis images, *p* <0.001; FWE-corrected in axial, coronal and sagittal and 3D views)

Supplemental Table 4. Brain regions implicated in the fMRI cannabis cue reactivity task across the whole sample (*p*<0.001, FWE corrected)

Cluster	Peak					MNI coordinates			AAL labels	
k	p(FWE)	p(FDR)	TFCE	Z	p(unc)	x	y	z		
**cannabis > neutral images**										
4226	<.001***	<.01**	14873.95	3.35	<.001***	-8	-52	28	Precuneus	left
	<.001***	<.01**	13874.41	3.35	<.001***	6	-52	26		right
	<.001***	<.01**	12723.94	3.35	<.001***	0	-60	34		left
5602	<.001***	<.01**	13552.96	3.35	<.001***	-6	52	-8	Medial OFC, left	
	<.001***	<.01**	13351.92	3.35	<.001***	-4	42	-2	ACC, left	
	<.001***	<.01**	12874.29	3.35	<.001***	-2	36	-8		
377	<.01**	<.01**	6584.78	3.35	<.001***	-26	-34	-6	Hippocampus, left	
	<.01**	<.01**	6441.6	3.35	<.001***	-24	-22	-16		
	<.01**	<.01**	5673.34	3.35	<.001***	-20	-38	2		
135	<.01**	<.01**	5996.43	3.35	<.001***	62	-16	32	Postcentral gyrus, right	
37	<.01**	<.01**	5190.96	3.35	<.001***	-30	6	-16	Amygdala, left	
	<.01**	<.01**	5124.96	3.35	<.001***	-34	14	-14	Insula cortex, left	
19	<.01**	<.01**	5168.55	3.35	<.001***	-42	10	-6		
6	<.01**	<.01**	5078.22	3.35	<.001***	28	-22	-14	Hippocampus, right	
10	<.01**	<.01**	5066.02	3.35	<.001***	-64	-8	-14	Middle temporal gyrus, left	
**neutral > cannabis images**										
748	<.001***	<.01**	2426.83	3.35	<.001***	14	-74	0	Lingual gyrus, right	
	<.001***	<.01**	2387.86	3.35	<.001***	8	-82	0		
	<.001***	<.01**	2315.09	3.35	<.001***	12	-82	10	Calcarine sulcus, right	
149	<.001***	<.01**	1640.99	3.35	<.001***	-38	6	30	Inferior frontal gyrus, operculum, left	
	<.01**	<.01**	1461.85	3.35	<.001***	-46	12	30		
77	<.01**	<.01**	1459.22	3.35	<.001***	-52	30	20		
	<.01**	<.01**	1430.49	3.35	<.001***	-44	20	26	Inferior frontal gyrus, triangularis, left	

Note: Superior medial OFC superior frontal gyrus medial orbital, left.

Abbreviations: k, cluster size; *p*, p-value; FWE, family-wise error; FDR, family discovery rate; TFCE, threshold-free cluster enhancement; Z, z-statistic; unc, uncorrected; MNI, Montreal Neurological Institute; AAL, Automated Anatomical Atlas; ACC, anterior cingulate cortex; OFC, orbitofrontal cortex.

***p < .001, **p < .01, *p < .05

8. Brain activity in the the *CUD and control groups, separately,* during cannabis cue-reactivity fMRI task

8.1. Brain activity in the the *CUD group*

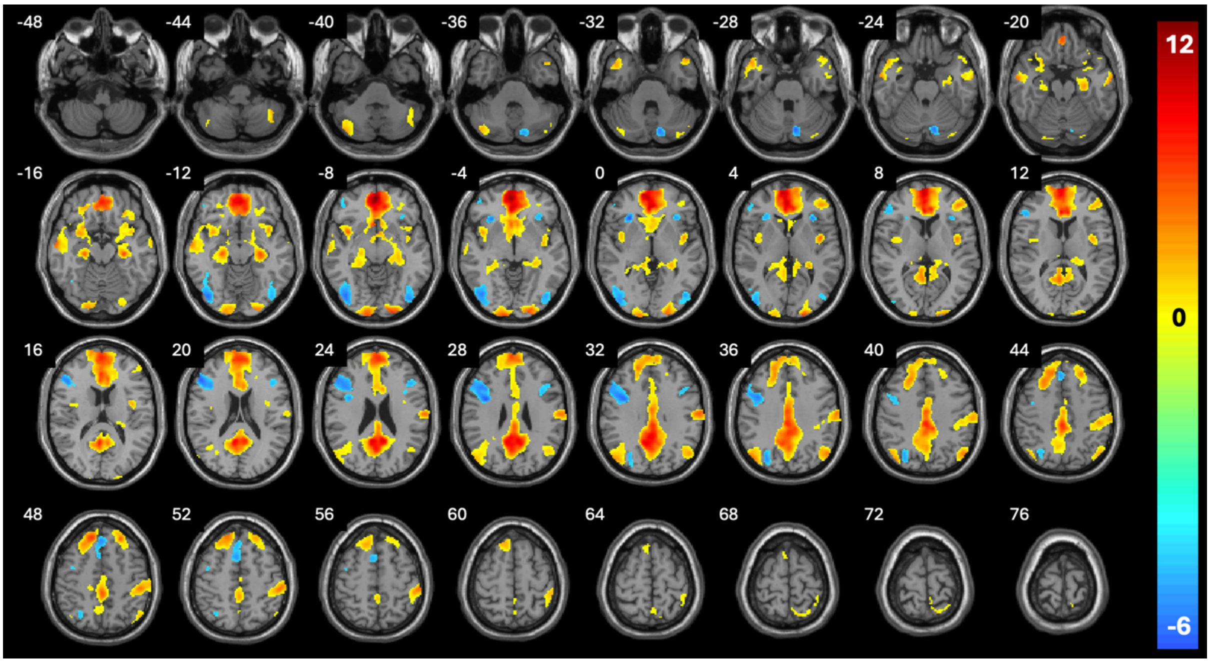

Supplemental Figure 2. **Axial slices (slice N on top left of slices), showing brain regions of differences for the *CUD group* during the contrasts (i) cannabis > neutral images, in yellow-to-red hues; (ii) cannabis<neutral images in yellow-to-blue hues. Cluster level FWE-corrected p value < 0.05, with initial uncorrected p < 0.001, k > 50.**

Supplemental Table 5. Brain regions implicated in the fMRI cannabis cue reactivity task in the *CUD* group (*p*<0.001, FWE corrected)

Cluster			Peak					
k	**p(FWE)**	**p(FDR)**	T	p(unc)	x	y	z	AAL Labels
**cannabis > neutral images**								
19497	<.001***	<.001***	13.17	<.001***	-4	52	-6	Medial OFC, left
			11.72	<.001***	-10	58	2	Superior medial frontal, left
			11.31	<.001***	-4	62	8	
693	<.001***	<.001***	8.49	<.001***	18	-98	-6	Calcarine sulcus, right
			8.47	<.001***	4	18	-102	Superior occipital, right
			4.70	<.001***	36	-82	-30	Cerebelum, Crus1, right
639	<.001***	<.001***	8.16	<.001***	-14	-102	-6	Calcarine sulcus, left
			6.54	<.001***	-20	-96	-14	Lingual gyrus, left
			5.61	<.001***	-12	-90	-16	
1644	<.001***	<.001***	7.68	<.001***	64	-14	34	Postcentral gyrus, right
			7.11	<.001***	54	-28	54	
			7.08	<.001***	62	-14	24	
566	<.001***	<.001***	7.40	<.001***	24	38	46	Superior frontal, right
			4.42	<.001***	28	28	52	
			3.92	<.001***	18	38	34	
839	<.001***	<.001***	6.88	<.001***	-48	-72	36	Mid occipital, left
			5.32	<.001***	-44	-68	44	Angular gyrus, left
			4.66	<.001***	-38	-56	30	
573	<.001***	<.001***	6.88	<.001***	50	-68	38	Angular gyrus, right
393	<.001***	<.001***	6.87	<.001***	42	44	6	Mid frontal, right
196	<.01**	<.01**	6.81	<.001***	60	-10	-22	Mid temporal, right
			4.49	<.001***	50	-4	-24	
			3.74	<.001***	54	8	-30	Mid temporal pole, right
147	<.05*	<.05*	6.30	<.001***	42	18	-34	
229	<.01**	<.01**	5.88	<.001***	-44	-74	-38	Cerebellum, Crus 2, left
			4.51	<.001***	-42	-66	-40	Cerebellum, Crus 2, left
122	<.05*	<.05*	5.62	<.001***	42	-50	-44	Cerebelum, lobule 7b, right
			5.34	<.001***	42	-62	-44	Cerebellum, Crus 2, right
			3.42	<.001***	48	-70	-34	Cerebellum, Crus 1, right
**cannabis < neutral images**								
764	<.001***	<.001***	-3.17	<.001***	-50	-64	0	Mid temporal, left
			-3.17	<.001***	-44	-72	4	
			-3.17	<.001***	-36	-84	-12	Inferior occipital, left
1420	<.001***	<.001***	-3.17	<.001***	-52	18	24	Inferior frontal gyrus, triangularis, left

			-3.17	<.001***	-50	22	36	Mid frontal, left
			-3.17	<.001***	-50	46	2	Inferior frontal gyrus, triangularis, left
134	<.05*	<.05*	-3.17	<.001***	-34	24	4	Insula, left
			-3.17	<.001***	-30	28	-6	Inferior frontal gyrus, pars orbitalis, right
			-3.23	<.001***	-26	26	4	Insula, left
348	<.001***	<.001***	-3.17	<.001***	42	-60	-12	inferior temporal, right
			-3.18	<.001***	44	-80	4	Occipital, mid right
			-3.18	<.001***	46	-72	-12	Inferior occipital, right
292	<.001***	<.001***	-3.17	<.001***	-26	-64	40	Mid occipital, left
			-3.18	<.001***	-24	-78	36	
			-3.18	<.001***	-28	-70	46	Inferior Parietal, left
134	<.05*	<.05*	-3.17	<.001***	36	28	2	Inferior frontal gyrus, pars triangularis, right
			-3.18	<.001***	36	24	10	
			-3.19	<.001***	34	28	-8	Inferior frontal gyrus, pars orbitalis, right
201	<.01**	<.01**	-3.18	<.001***	46	20	28	Inferior frontal gyrus, pars triangularis, right
			-3.18	<.001***	36	10	32	Inferior frontal gyrus, operculum, right
			-3.19	<.001***	38	16	24	Inferior frontal gyrus, pars triangularis, right
239	<.01**	<.01**	-3.18	<.001***	4	-76	-26	Cerebellum, vermis 7
			-3.20	<.001***	10	-70	-30	
			-3.22	<.001***	12	-76	-20	Cerebellum, lobule 6, right
336	<.001***	<.001***	-3.18	<.001***	4	36	46	Medial superior frontal, right
			-3.19	<.001***	0	12	50	Supplementary Motor Area, left
			-3.19	<.001***	-6	24	54	

N.B. Cluster level FWE-corrected p value < 0.05, with initial uncorrected p < 0.001, k > 50.

***p < .001, **p < .01, *p < .05.

8.2. Brain activity in the the *control group* during cannabis cue-reactivity fMRI task

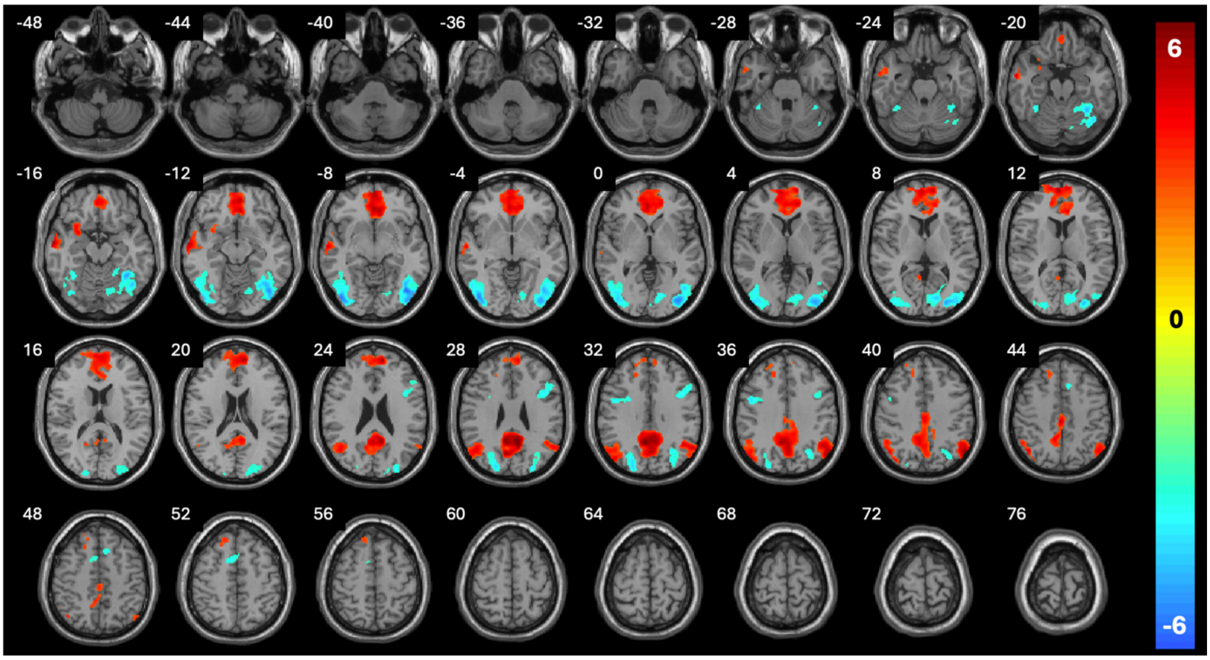

Supplemental Figure 3. **Axial slices (slice N on top left of slices), showing brain regions of differences for the *control group* during the contrasts (i) cannabis > neutral images, in yellow-to-red hues; (ii) cannabis<neutral images in yellow-to-blue hues. Cluster level FWE-corrected p value < 0.05, with initial uncorrected p < 0.001, k > 50.**

Supplemental Table 6. Brain regions implicated in the fMRI cannabis cue reactivity task in the *control* group (*p*<0.001, FWE corrected)

Cluster			Peak					
k	**p(FWE)**	**p(FDR)**	T	p(unc)	x	y	z	AAL Labels
**cannabis > neutral images**								
2598	<.001***	<.001***	7.17	<.001***	6	-52	28	Precuneus, right
			7.02	<.001***	-8	-52	30	Posterior cingulum, left
			6.31	<.001***	-8	-66	28	Cuneus, left
699	<.001***	<.001***	6.77	<.001***	52	-66	38	Angular gyrus, right
			5.72	<.001***	48	-56	36	
			5.4	<.001***	54	-60	32	
4087	<.001***	<.001***	6.6	<.001***	10	56	22	Medial superior frontal, right
			6.22	<.001***	-6	62	12	Medial superior frontal, left
			6.18	<.001***	12	62	6	Medial superior frontal, right
432	<.001***	<.001***	6.47	<.001***	-62	-8	-14	Mid temporal, left
			4.01	<.001***	-56	4	-30	
			3.92	<.001***	-56	4	-10	Superior temporal, left
794	<.001***	<.001***	5.52	<.001***	-48	-74	34	Angular gyrus, left
			5.32	<.001***	-46	-62	26	
			5.22	<.001***	-46	-64	44	
127	<.05*	<.05*	5.16	<.001***	-30	6	-16	Amygdala, left
			4.73	<.001***	-32	14	-16	Insula, left
195	<.01**	<.01**	4.3	<.001***	-16	36	52	Superior frontal, left
			4.25	<.001***	-18	36	34	
			3.65	<.001***	-20	26	48	Mid frontal, left
**cannabis < neutral images**								
326	<.001***	<.001***	-3.17	<.001***	52	24	32	Mid frontal, right
			-3.17	<.001***	38	16	30	Inferior frontal gyrus, pars opercularis, right
			-3.17	<.001***	44	26	26	Inferior frontal gyrus, pars triangularis, right
3359	<.001***	<.001***	-3.17	<.001***	42	-54	-14	Inferior temporal, right
			-3.17	<.001***	28	-76	28	Mid occipital, right
			-3.17	<.001***	24	-86	14	Superior occipital, right
162	<.05*	<.05*	-3.17	<.001***	2	18	52	Supplementary motor area, right
			-3.17	<.001***	10	22	50	
			-3.18	<.001***	-8	8	56	Supplementary motor area, left
1671	<.001***	<.001***	-3.17	<.001***	-40	-48	-16	Inferior temporal, left
			-3.17	<.001***	-30	-90	14	Occipital, mid left
			-3.17	<.001***	-38	-84	12	
328	<.001***	<.001***	-3.17	<.001***	-30	-82	34	
			-3.18	<.001***	-26	-82	42	

			-3.19	<.001***	-20	-90	26	Superior occipital, left
166	<.05*	<.05*	-3.17	<.001***	-32	8	30	Precentral gyrus, left
			-3.18	<.001***	-42	2	30	
			-3.19	<.001***	-28	2	34	

N.B. Cluster level FWE-corrected p value < 0.05, with initial uncorrected p < 0.001, k > 50.

***p < .001, **p < .01, *p < .05

9. Additional results on correlations before and after outlier removal

9.1 Descriptives for participants who met criteria for outliers

Sensitivity brain-behavior correlations were run in the whole sample before and after outlier removal. Outliers are described below as a function of the relevant variable.

There were 6 outliers, for alcohol standard drinks/past month. They included 2 participants with moderate CUD (i.e., 206.8 drinks in a male aged 43; 201 drinks in a female aged 20); and 4 participants with severe CUD (i.e., 156.4 drinks in a 29 year old female; 150.9 drinks in a 36 year old male; 111.4 drinks in a male aged 29; and 101.4 drinks in a male aged 39). Duration of abstinence from cannabis use showed 8 outliers. Of these, one endorsed a moderate CUD (i.e., 64.5 hours in a male aged 24). The remainder had severe CUD and included 6 males (i.e., age 26, 73.25 hours; age 30, 44 hours; age 22, 44 hours; age 20, 40.5 hours; age 29, 38.25 hours) and 2 females (i.e., age 21, 38.25 hours; and age 18, 48 hours). Outliers also included a male with moderate CUD aged 34, with a BDI score of 46; and a female with severe CUD aged 22, with β values for middle occipital gyrus being 2.60.

9.2 Correlations that were significant before and after outlier removal

Within the CUD group, the following correlations were significant after outlier removal. These included: positive correlations between greater superior occipital cortex activity and arousal ratings of cannabis (*minus*) neutral images (VAS scores; rho = 0.40, $p < .05$), and withdrawal (CWS scores; rho = 0.31, $p < .05$); negative correlations between THC-

COOH:creatinine levels & activities of the ACC and inferior parietal cortices (rho = -0.33, $p < .05$ and rho = -0.33, $p <.05$, respectively).

9.3 Correlations that were significant in the whole sample & that did not survive outlier removal

In the whole group of participants with a CUD, the following correlations were significant in the whole sample, but did not survive outlier removal. There were significant negative correlations between lower THC-COOH:creatinine levels and greater postcentral/supramarginal activity (rho = -0.30, $p < .05$ and rho = -0.28, $p <.05$, respectively); and positive correlations between greater middle occipital/cerebellum activity and withdrawal (CWS; rho = 0.33, $p < .05$ and rho = 0.34, $p <.05$, respectively) and positive correlations between the calcarine sulcus, middle occipital gyrus, and superior occipital gyrus with depression symptoms (BDI; rho = 0.29, $p < .05$ and rho = 0.34, $p <.05$, rho = 0.30, $p <.05$ respectively). After outlier removal, none of the results were significant.

10 Exploring the relationship between COVID-related stress and cannabis use

Supplemental Table 7. Spearman's correlations between cannabis use metrics and COVID-stress subscales in CUD participants (rho, n, *p*).

	Cannabis use metrics	
**COVID-Stress subscales**	**Dosage/past month (grams)**	**Days/past month**
**Danger fears**	-0.12, 49, .41	-0.08, 49, .57
**Socioeconomic consequences**	0.09, 49, .52	0.06, 49, .69
**Xenophobia**	0.04, 49, .76	0.03, 49, .82
**Contamination fears**	-0.10, 49, .48	-0.04, 49, .78
**Traumatic stress**	-0.12, 49, .43	0.03, 49, .84
**Compulsive checking and reassurance seeking**	0.10, 49, .49	0.17, 49, .25

11 References

1. Babor TF, Higgins-Biddle JC, Saunders JB, Monteiro MG, Organization WH (2001): AUDIT: The alcohol use disorders identification test: Guidelines for use in primary health care. World Health Organization.

2. Lorenzetti V, Solowij N, Whittle S, Fornito A, Lubman DI, Pantelis C, Yücel M (2015): Gross morphological brain changes with chronic, heavy cannabis use. *Br J Psychiatry*. 206:77-78.

3. Solowij N, Stephens RS, Roffman RA, Babor T, Kadden R, Miller M, et al. (2002): Cognitive functioning of long-term heavy cannabis users seeking treatment. *JAMA*. 287:1123-1131.

4. Yucel M, Solowij N, Respondek C, Whittle S, Fornito A, Pantelis C, Lubman DI (2008): Regional brain abnormalities associated with long-term heavy cannabis use. *Arch Gen Psychiatry*. 65:694-701.

5. Onaemo VN, Fawehinmi TO, D'Arcy C (2021): Comorbid Cannabis Use Disorder with Major Depression and Generalized Anxiety Disorder: A Systematic Review with Meta-analysis of Nationally Representative Epidemiological Surveys. *J Affect Disord*. 281:467-475.

6. Wechsler D (2011): Wechsler Abbreviated Scale of Intelligence-(WASI-II). Bloomington, MN: NCS Pearson. *Inc[Google Scholar]*.

7. First MB, Williams JB, Karg RS, Spitzer RL (2015): Structured clinical interview for DSM-5—Research version (SCID-5 for DSM-5, research version; SCID-5-RV). *Arlington, VA: American Psychiatric Association*. 2015:1-94.

8. Allsop DJ, Norberg MM, Copeland J, Fu S, Budney AJ (2011): The Cannabis Withdrawal Scale development: patterns and predictors of cannabis withdrawal and distress. *Drug Alc Depend*. 119:123-129.

9. Lorenzetti V, Solowij N, Whittle S, Fornito A, Lubman DI, Pantelis C, Yücel M (2015): Gross morphological brain changes with chronic, heavy cannabis use. *The British Journal of Psychiatry*. 206:77-78.

10. Sobell LC, Sobell MB (1992): *Timeline Follow-Back: A technique for assessing self-reported alcohol consumption*. Totowa NJ: Humana Press.

11. Fagerstrom K, Russ C, Yu CR, Yunis C, Foulds J (2012): The Fagerstrom Test for Nicotine Dependence as a predictor of smoking abstinence: a pooled analysis of varenicline clinical trial data. *Nicotine Tob Res*. 14:1467-1473.

12. Beck AT, Steer RA, Brown GK (1996): *Manual for the Beck Depression Inventory-II*. San Antonio, TX: Psychological Corporation.

13. Spielberger CD, Gorsuch RL (1983): *Manual for the State-Trait Anxiety Inventory, Form Y ("self-evaluation questionnaire")*. Palo Alto, California: Consulting Psychologists Press.

14. Cohen S, Kamarck T, Mermelstein R (1983): A global measure of perceived stress. *J Health Soc Behav*. 24:385-396.

15. Konings M, Bak M, Hanssen M, Van Os J, Krabbendam L (2006): Validity and reliability of the CAPE: a self-report instrument for the measurement of psychotic experiences in the general population. *Acta Psychiatrica Scandinavica*. 114:55-61.

16. Taylor S, Landry CA, Paluszek MM, Fergus TA, McKay D, Asmundson GJG (2020): Development and initial validation of the COVID Stress Scales. *J Anxiety Disord*. 72:102232.

17. Cousijn J, Watson P, Koenders L, Vingerhoets W, Goudriaan A, Wiers R (2013): Cannabis dependence, cognitive control and attentional bias for cannabis words. *Addictive behaviors*. 38:2825-2832.

18. Esteban O, Markiewicz CJ, Blair RW, Moodie CA, Isik AI, Erramuzpe A, et al. (2019): fMRIPrep: a robust preprocessing pipeline for functional MRI. *Nature Methods*. 16:111-116.

19. Parkes L, Fulcher B, Yücel M, Fornito A (2018): An evaluation of the efficacy, reliability, and sensitivity of motion correction strategies for resting-state functional MRI. *Neuroimage*. 171:415-436.
